# Automotive IoT Ethernet-Based Communication Technologies Applied in a V2X Context via a Multi-Protocol Gateway

**DOI:** 10.3390/s22176382

**Published:** 2022-08-24

**Authors:** Alexandru Ioana, Adrian Korodi, Ioan Silea

**Affiliations:** Department of Automation and Applied Informatics, Faculty of Automation and Computers, University Politehnica Timișoara, 300223 Timisoara, Romania

**Keywords:** automotive, communication protocols, DDS, eCAL, interoperability, IoT, multi-protocol gateway, SOME/IP

## Abstract

The architectural approach for complex communication systems must adapt quickly and take into consideration the increasing set of requirements for every industrial field. The automotive domain is evolving toward the electrification era, with massive technological transformations being realized on all architectural, hardware, and software levels. The legacy usage of exclusively microcontrollers is altered by adopting microprocessors with extended functionalities, reshaping the development structure. Although new hardware capabilities are available and Ethernet communication protocols can contribute to a new range of use-cases for intra-car or for vehicle-to-X (V2X) communication, the implications of using multiple protocols that cover different types of requirements, in the same architecture, are not fully determined. The importance of establishing clear expectations for intelligent communication systems considering various technological and architectural factors is significant for future improvements. In the current paper, we examine the compatibility and real-time responsiveness capabilities, in a diverse, service-oriented architecture, for the major automotive IoT Ethernet-based communication technologies. The feasibility analysis is materialized in a multi-protocol gateway solution that facilitates data exchange between entities with different technological origins. Scalable Service-Oriented Middleware over IP (SOME/IP) is considered the relevant protocol in the automotive domain, alongside the Data Distribution Service (DDS), which combines automotive and IoT applicability. The enhanced Communication Abstraction Layer (eCAL) middleware is added to the mix as an alternative solution for future communication scenarios. The obtained results confirm the compatibility between the targeted technologies, offering a clear understanding regarding the limits of a complex multi-protocol communication system. The defined service-oriented architecture offers efficient data exchanges in a gateway context, also allowing the exploration of the real-time capabilities.

## 1. Introduction

The expansion towards Industrial Internet of Things (IIoT) and Industry 4.0 is shaping a new vision for the communication capabilities of the current industrial systems [1]. The objectives regarding the feasibility and flexibility for each technological enhancement remain and are even complemented with additional requirements and demands. The transition to the next phase is slow but constant. In recent years, the progress of Ethernet-based communication technologies has had a significant impact on the already acknowledged methods of development, opening new possibilities and identifying new needs and challenges on the way. The huge amount of data exchanged across distributed architectures containing different types of devices and entities, at high rates, in a deterministic and automatic manner, can be established as the main step necessary for the evolution toward complex interconnected industrial systems. Although at first it might appear different, the interconnectivity concept is relevant and applicable to all the industrial fields. As the ability to interface more complex systems will increase, whether the concept applies in the manufacturing domain, power grids, industrial water stations, cloud infrastructures, or the automotive field, the capabilities will grow and contribute to increased productivity, quality, and efficiency [2,3]. The impact of such a transition will be felt also with the emergence of new business cases and strategies, and administrative cost reduction for multiple industrial processes.

The Ethernet communication protocols are more present in industrial systems and are compliant with interoperability and flexibility standards, offering multiple solutions for IoT scenarios. However, in the case of real-time behavior for field devices involved in industrial processes, the expectations are not clearly defined, mainly due to the multitude of factors that can influence the performance and feasibility of Ethernet-based communication structures. From an academic perspective, there is a need for practical studies regarding architectures and prototypes that address the ongoing concerns, studies that can provide a concrete set of expectations and a feasible starting point for future industrial applications.

The automotive domain has already begun a transformation phase from combustion-based vehicles toward the electrification era. Such a significant transition will imply massive changes in the architectural, hardware, and software levels and can also establish the technological foundation for completely integrating the automotive field in the IoT/IIoT. With expanding visions such as autonomous driving [4], software-defined vehicles, alongside the refinement and improvement of the AUTOSAR standard, in the coming years, the expectations are high for the key players in the market, and Ethernet-based communication capabilities will play a significant role in achieving the objectives.

Although the industry transition has begun and new approaches are considered, it must remain clear that many already-established technologies specific to the automotive domain will carry on and will also be a contributing factor to future advancements. The transformation itself should be interpreted as an enriched strategy going further with multiple communication protocols such as CAN, CAN-FD, LIN, FlexRay, and others still having a wide scope for multiple purposes. It remains to be seen if the area of applicability for such technologies will remain the same, or if changes will happen on the way. In [5], the authors mention that “FlexRay, Control Area Network (CAN), Local Interconnect Network (LIN), CAN-FD, Automotive Ethernet are the principal automotive communication protocols used in today’s Electric and Electronic Vehicle Architectures (E/E architecture)”. From the AUTOSAR perspective, the interpretation should be similar, even if the Adaptive AUTOSAR side is continuously growing and has a more accentuated impact than in the past. The importance of having an adopted industrial standard is related to the insight that all the available and validated concepts will remain in use, and the evolution of the standard will dictate the pace of the progress. In the short term, other industries may benefit faster from new technological capabilities, and this is the main reason why other industries have a better-defined direction towards IIoT and Industry 4.0 (e.g., Open Platform Communication Unified Architecture, OPC UA, is a key enabler [6] for Industry 4.0, and even the AUTOSAR-compliant Data Distribution Service is gaining applicability in manufacturing [7]). However, in the long term, the stability and sustainability generated by the AUTOSAR standard provide considerable potential for the automotive business in the future and could represent the differences in later advancements. 

The current state of the automotive field and the available and compliant communication solutions facilitate a new research direction. This direction considers the adoption of multiple Ethernet-based communication protocols to obtain enhanced systems that are able to extend the applicability to V2X scenarios and also to increase the efficiency of intra-car communication procedures. From an academic perspective, such an approach has the potential to extend the knowledge of Ethernet communication, maintaining the high-performing and time deterministic objectives important to the automotive domain, and at the same time, aligning with the new envisioned architectures specific to the electrification era. 

The growing influential presence of microprocessors in vehicle architectures, alongside Adaptive AUTOSAR and POSIX-based operating systems, is offering the opportunity to define new concepts, and from the communication perspective, to analyze new technologies in an industrial context. This will showcase advantages and disadvantages in a concrete manner. The current paper provides insights regarding the state-of-the-art communication protocols compliant with the automotive demands, and also aims to extend the perception towards IoT/IIoT, developing a gateway solution based on AUTOSAR-compliant Ethernet-based communication technologies: Scalable Service-Oriented Middleware over IP (SOME/IP) and Data Distribution Service (DDS) in the same context with another emerging middleware solution, Enhanced Communication Abstraction Layer (eCAL).

Given the above-mentioned context, the current work aims to:

- Analyze the compatibility of the selected communication technologies, combining strategies and visions with high potential and highlighting advantages and disadvantages from multiple perspectives, allowing a better understanding of how each protocol can be used in the right context.
- Define an appropriate architecture aligned with the latest adaptive architectural principles that includes necessary elements for increased applicability in automotive and IIoT applications, and addresses challenges specific to Ethernet technologies.
- Conceive and develop a configurable multi-protocol gateway solution that can interface completely separated SOME/IP, DDS, and eCAL entities, expanding the applicability towards V2X concepts and interactions between communication structures specific to different industrial fields.
- Explore real-time capabilities of the multi-protocol gateway solution, examining the possible impact of future achievable improvements and establishing a clear set of expectations concerning the evolving industrial requirements.

Section 2 presents an automotive applicability study of the proposed concept. Section 3 provides in-depth details for each communication technology, approaching concepts of interest for enhanced interoperability use-cases. Section 4 describes the defined system architecture with the associated particularities, offering a wider view of the scalability and complexity of the current work. Section 5 discusses the case studies, and based on relevant criteria and methods, presents a set of quantifiable results regarding the performance of the multi-protocol gateway solution. Section 6 offers insights on the encountered challenges, suggesting future development directions and concise conclusions.

## 2. Automotive Applicability Study

**Figure 1:**
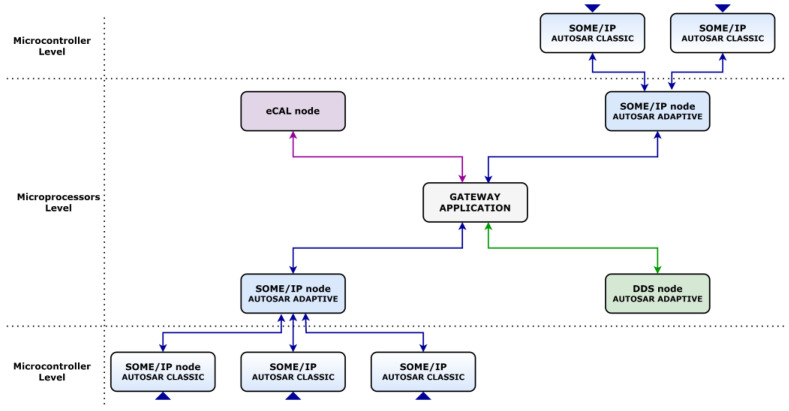
First industrial applicability perspective for the multi-protocol gateway.

In recent years, the in-vehicle Ethernet-based communication became more present, and the current transition to the electrification era will enhance its potential even further. It is expected for future architectures to contain communication nodes based on different protocols and middleware solutions, with various requirements and capabilities. Considering the importance of the AUTOSAR standard, it is safe to assume that some of the main challenges will concern the integration of standard compliant and non-compliant technologies and concepts in a modular manner, maintaining the principles and expectations of SOAs. The presence of various types of devices, technologies, and compliances will significantly slow the development process, so any feasible research concept needs to offer a clear applicability overview for the industry. The implemented gateway application deals with SOME/IP, DDS, and eCAL as communication technologies, assuring data exchange between independent nodes of the system. Considering the industrial context concerning the targeted middleware solutions, there are two different perspectives regarding how the concept can be applied successfully. The first perspective showcases that different SOME/IP, DDS, and eCAL nodes can interact by utilizing the gateway application as a central node for the data propagated from microcontrollers, in charge of different functional areas, to microprocessors that can supervise and manage data from multiple sources. In this context, the connectivity and interoperability between different protocols, devices and functional areas are assured, offering a clear solution for efficient in-vehicle communication. This first applicability perspective can be observed in Figure 1.

**Figure 2:**
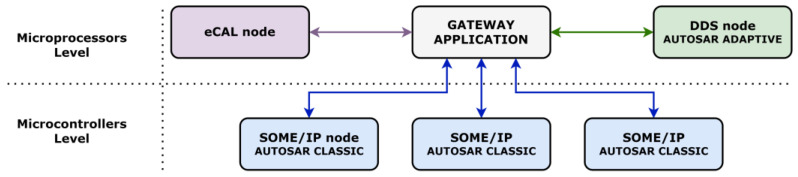
Second industrial applicability perspective for the gateway solution.

The second applicability perspective relies on the placement of the gateway application directly between classic, adaptive, and non AUTOSAR-compliant entities. Facilitating the data exchange between microcontrollers focused on individual functional areas and DDS and eCAL nodes, the multi-protocol gateway approach can overcome technological disadvantages related to the standard. For example, DDS being available for the adaptive platform and eCAL not being supported by the standard could still communicate with SOME/IP microcontrollers in charge of hard real-time procedures. This allows DDS and eCAL nodes to access the necessary information in a particular IoT or V2X scenario or supervise the activity on designated functional areas without support on the classic platform. This second applicability perspective can be observed in Figure 2.

## 3. Tools and Technologies in the Current State-of-the-Art Context

This section focuses on technical details regarding SOME/IP, DDS, and eCAL communication middleware with respect to specific standards and approaches and relevant state-of-the-art context.

### 3.1. Scalable Service-Oriented Middleware over IP (SOME/IP)

In recent years, the complexity and diversity of requirements increased significantly in the automotive field. This growth resembles, to some extent, similar evolutions from different industries, such as manufacturing, industrial robotics, industrial plants, telematics, and others. Although the objectives behind this process had a different, more domain-oriented origin, the outcome has prepared state-of-the art technologies for a complex set of usage scenarios. The need to use cameras, complex sensors, machine learning and advanced security mechanisms, the need to connect to non-AUTOSAR components, the integration of end-user equipment, and other procedures have influenced the expansion of Service-Oriented Architectures (SOA) across the majority of automotive areas. SOAs assure the exchange of large and complex sets of data between providers and consumers, combining the client–server and publish–subscribe paradigms in a decoupled and abstract manner. Alongside the increased flexibility, having the information distributed as designated services allows enhanced architectural designs blending different types of hardware and technologies and aligning with both the classic and adaptive sides of the standard. 

SOME/IP is designed to be compliant with the AUTOSAR standard and provides all the necessary mechanisms to map the SOA concept. It is integrated into both classic and adaptive sides, assuring a high quality of service (QoS) for communication scenarios. From a security perspective, in [8], the authors conclude the lack of protocol-embedded security because it has been designed to operate on top of classical transport protocols, and they propose a framework to secure the SOME/IP middleware. A solution that maintains the network capabilities of the protocol is provided in [9]. Ref. [10] presents a security analysis, evaluating the attack possibilities of SOME/IP in the publish–subscribe design, and proposes enhanced security extensions. In the context of enlarging the use of SOME/IP, the security is approached also in other works (focusing on in-vehicle Ethernet-based network security [11], or general challenges [12]). Although there are emerging studies on the security topic for SOME/IP, the subject is of great interest. At the moment, the approaches mainly derive from the automotive standpoint based on current use-cases. However, in the future, considering the rapid expansion and the potential of the protocol, new security measures might be needed for more diverse scenarios.

SOME/IP is part of the communication management API on Adaptive AUTOSAR, which can be viewed as a more abstract layer that deals with communication concepts for all the middleware technologies that are supported in the standard. Following the principles of SOA, the communication management classifies services into three distinct categories: methods, events, and fields. Methods can be depicted as services based on the classic client–server design, with request and response procedures between involved entities. Events are based on the publish–subscribe design, the data being delivered either cyclically or sporadically between dedicated providers and consumers. Fields can be described as a combination of methods and events, offering, besides cyclical deliveries, the option to access the data via get and set methods [13,14,15]. Although the majority of the Ethernet-based protocols support the client–server design and the real-time-oriented publish–subscribe design, in complex industrial scenarios, it is expected that a mix of the two designs would actually be the most efficient solution. The handling of the services in an abstract manner, regardless of the communication protocol used for transmission, can be considered a powerful mechanism, especially for diverse architectures and requirements. In Ref. [16], the authors explain the current trends in automotive E/E architectures, emphasizing the presence of centralized servers for smart actuators and real-time control procedures. One of the highlighted practical solutions for architectural simplification is the unification of functionally related subdomains (e.g., Powertrain and Chassis). With the appearance of high-performance computers (as servers), the manageable use-cases are numerous. Although SOME/IP fulfils the needs regarding resource consumption, compatibility, and scalability [17], the requirements will evolve proportionally with the hardware capabilities. Refs. [18,19] are approaching the SOME/IP in the context of interacting with the local in-vehicle legacy communication solutions. In [20], the authors are approaching fault tolerance and redundancy using SOME/IP middleware as needed for current safety requirements in the automotive domain, mentioning that the Adaptive AUTOSAR standard does not specify requirements regarding fault tolerance. 

Due to its high reliability and applicability in the automotive domain, SOME/IP was considered a suitable candidate also for vehicle-to-infrastructure scenarios, and recent studies with practical focus have emerged. In [21,22], a gateway between SOME/IP and OPC-UA protocol was approached, targeting the real-time capabilities in an IoT scenario. The results confirmed the compatibility of the protocols in multiple architectures and designs. With OPC-UA as one of the most utilized technologies, future possible interactions with automotive technologies in an AUTOSAR-based scenario need to be taken in consideration. In Ref. [23], an architecture is defined for the binding between the adaptive platform and OPC-UA, and the prototype validates the capabilities of OPC-UA as a middleware solution for service-oriented communication in AUTOSAR. The validation of automotive technologies in industrial and IoT scenarios, and of industrial-specific protocols in AUTOSAR-compliant scenarios, concludes that the next step for enhanced interconnectivity will be based on efficient interactions between the most capable and utilized communication protocols from various industries.

At present, SOME/IP is widely used by all the important players of the automotive market, being validated in multiple designs and architectures [24]. Although it satisfies the expectations for now, the rapid growth of the requirements, the complexity of future network topologies, and other available solutions should extend the vision toward mixed nodes based on different communication technologies inside the same architecture. In such a case, the link between the nodes with different technological origins is represented by advanced gateway applications that facilitate the interactions.

### 3.2. Data Distribution Service (DDS)

Following the industrial model regarding the transition to Industry 4.0, it is clear that the next steps will heavily focus on real-time communication and architectural refinements for enhanced capabilities, also targeting cost reductions at each development stage. A significant impact, derived from the fast transformation of current industrial systems, is experienced in the evolution of general requirements. Due to such a dynamic context, the Ethernet-based communication became the backbone of intelligent systems, and the cross-domain interactions between legacy protocols are the subject of emerging studies.

The DDS middleware offers a publish–subscribe design, oriented to time deterministic applications, and a classic request–reply design for remote procedure calls, enabling efficient data distribution among participants. Besides high performance and predictability, DDS offers a set of mechanisms designated for achieving different types of objectives, assuring an efficient use of resources. For completely separating functional areas inside an architecture, the system can be split into multiple domains, each one dealing only with specific data and interested consumers and providers. The joining entities are referred to as domain participants, and the data exchange is performed in a designated virtual space inside the system. This type of mechanism enhances the flexibility that DDS presents, allowing a manageable design even for a large system. Another benefit derived from having separated virtual spaces for different functionalities is the ability of adding or removing functionalities to a complex architecture in a granular way. As some of the challenges for future cross-domain applications are represented by the high number of domain-specific dependencies and increased software complexity, an advantage related to modular management can be decisive when a communication technology is taken into consideration.

The information exchanged between publishers and subscribers is structured as topics, and every interested consumer can subscribe to one or more topics. Each topic can have multiple instances, and through data readers and writers, the information is updated and distributed efficiently. In general, the topics are based on complex data types that can be defined using the available integrated mechanisms, allowing a clear structuring of the payload before delivery.

With available implementations from vendors, DDS compatibility and portability are assured by using Real-Time Publish–Subscribe (RTPS) as wire protocol. At the transport level, TCP or UDP are used, the DDS subcomponents and features being mapped on top of RTPS protocol. The advantage of accessing all DDS mechanisms without dealing with constraints and dependencies regarding the provided technology stack is decisive for dynamic systems with different types of requirements. From a security perspective, in SOAs, the general goals are clear: authentication and authorization for each participant, integrity and confidentiality for the distributed data, cryptographic support mechanisms, and message origin authentication. In case of DDS, a collection of security features is available, alongside the possibility to extend capabilities using plug-in interfaces. However, recent analyses of DDS and available implementations revealed a set of exploitable vulnerabilities. The Industrial Control System Advisory addressed the matter, evaluating the risks and providing vulnerability early notice and identifying baseline mitigations [25]. DDS security issues are approached in [26].

As for SOME/IP, DDS is also bound to the communication management cluster on Adaptive AUTOSAR, confirming compliance with the standard and with the automotive field. Although it is supported as a communication middleware in the standard, DDS is not widely used by the majority of leading companies on the market. Only in recent years, it began to attract more attention as the industry needs have expanded to heavy interoperability use-cases. The protocol is approached for autonomous vehicles [27,28], aerial vehicle formation control [29], and other real-time multi-processor distributed systems [30]. As the protocol is already supported by the adaptive platform, for a feasible increase in automotive applicability, objectives toward DDS support in the classic platform must be set. Even with the acknowledgement of a more frequent presence of adaptive applications in today’s development process, for a realistic integration in future SOAs, the communication technologies must be supported by both the classic and adaptive platforms. As part of the communication management API, the conceptual approach of methods, events, and fields is also applied in the case of DDS, offering the same standardized strategy for implementing adaptive applications, regardless of which middleware is used.

From an academic standpoint, with IoT as a subject of high interest for the researchers and for the industry, DDS is representing a high potential technology [31], and studies are focusing on finding the most proficient communication protocols that can satisfy the growing set of requirements. With DDS as the communication middleware for ROS2 (Robot Operating System 2), an open source framework designated for advanced control of industrial robots, multiple studies have evaluated the capabilities and the real-time behavior of the protocol [32,33,34,35]. In [36,37,38], DDS is used alongside other IoT-specific protocols, proving to be a flexible and scalable solution according to industrial standards. In Ref. [39], the authors define a suitable architecture for coexistence and gateway communication between DDS and OPC-UA, analyzing the real-time responsiveness of the protocols on field devices. 

At the moment, DDS is present in research and industry, being validated in a wide range of scenarios. The compliance with the AUTOSAR adaptive platform allows new possible strategies for future automotive products in terms of interconnectivity and interoperability, mapping on the technological upgrades generated by the transition to the electrification era. In coexistence with other communication technologies, with each DDS entity as part of a separated virtual space accessible only by its participants, the network topology difficulties can be overcome with less effort, making the system more flexible and scalable. Fulfilling the industrial criteria for numerous use-cases and considering the fast pace regarding the evolution of SOAs and requirements alongside the IIoT context, DDS can represent a feasible solution for complex systems with mixed technologies and demands. 

### 3.3. Enhanced Communication Abstraction Layer (eCAL)

The low latency data exchange solutions are needed in the context of next-level communication scenarios. The enormous amount of data, the complex configuration procedures, and hardware and operating system incompatibilities constitute major impediments in today’s development process. Besides the fundamental communication concepts offered, the ability to trace and visualize the distribution of messages with high priority, by a middleware solution, can be beneficial in complex architectures with a high number of nodes. The eCAL is an open source solution with automotive origins, designed for high-performance communication between different nodes with minimum configuration required. The protocol implements the publish–subscribe and client–server patterns following the same desired principles and mechanisms for industry demands. Considering the importance of industrial robot control use-cases in the IoT context, there is an available implementation of eCAL ROS2 as a layer with access to monitoring instruments. From a compatibility standpoint, there are stable eCAL versions that run on Linux, QNX, and Windows, supporting Intel and ARM platforms. As a fast, reliable, and scalable solution, assuring the conceptual needs of present communication structures, eCAL can be considered a viable protocol in an SOA. Due to high compatibility, low-resource usage, and easy configuration, eCAL can be perceived as a potential technology for both automotive and IoT purposes and should be examined in various circumstances for determining the most impactful applicability area.

### 3.4. Selected Context of Related Work

The context targeted for the ideal usage of the multi-protocol gateway is a V2X scenario. The current study focuses not only on automotive-related technologies but also on a capable IoT middleware. The V2X paradigm represents a high interest topic in recent years, with studies suggesting strategies and expanding and refining the concept at a fast pace. In Ref. [40], the authors highlight the research gaps regarding the autonomous driving concept, suggesting that future intelligent vehicles will heavily rely on measurement data from other participating vehicles under a V2V communication model. In [41], a three-layer architecture for vehicular IoT is suggested, with the middle layer being the network of interconnected smart devices, gateways, and servers needed to process and deliver the data. One of the vehicular IoT open issues highlighted by the authors is the “communication heterogeneity”, describing how challenging it is to interoperate “different entities that employ different communication protocols, and generate different data types”. The multi-protocol gateway addresses the efficient data exchange between entities based on different communication protocols, and the defined architecture aligns with the principles described by the research community, contributing to overcoming actual and acknowledged issues. In Ref. [42], the presence of a significant amount of real-time data acquired from sensors and actuators is anticipated for the autonomous vehicles, data that “must be processed and evaluated in order to receive timely decisions to benefit users”. A major objective of the study is targeting the real-time responsiveness of the system, allowing a deep understanding of the actual capabilities. Taking into account the concerns of the academic community and the industry dynamics, the implemented concept combines automotive and IoT principles in an innovative way, confirming the applicability in a V2X context.

With acknowledgement of all technical particularities of SOME/IP, DDS, and eCAL, a gateway scenario for a combined automotive and IoT approach can showcase how the advanced technologies should shape the next steps in a practical way. The GENIVI open-source implementation was used [43] for the development of all SOME/IP entities in the current system. For the implementation of DDS components, the eProsima Fast DDS [44] open source Software Development Kit (SDK) was used, alongside the eCAL middleware [45].

## 4. System Architecture

As state-of-the-art communication protocols are already established, a major obstacle to achieve interoperability in the future will consist of interfacing major technologies and architectures. Each one contains different types of hardware resources and addresses different types of requirements. For the current work, based on a combined automotive–IoT use-case, the initial focus targeted the definition of a suitable architecture with mixed SOME/IP, DDS, and eCAL nodes that could communicate inside the same network via a gateway, allowing the monitoring of the distributed data in a time deterministic manner.

**Figure 3:**
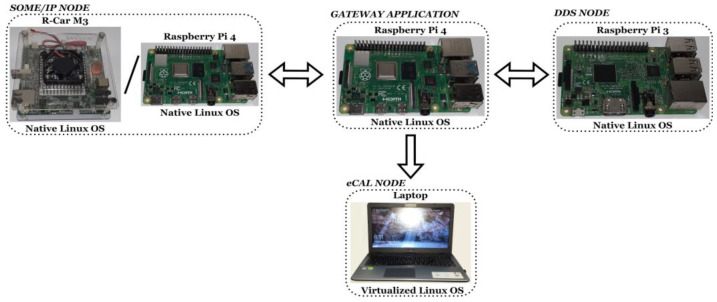
Hardware architecture.

From the hardware perspective, as a system with automotive-specific technologies, the architectural approach was oriented to the use of microprocessors with native POSIX-based operating systems for the majority of nodes. For a simulated interaction with an IoT supervisor node, the approach considered the use of a virtualized Linux OS on a general purpose computer. SOME/IP and DDS nodes can be viewed as the intra-car communication infrastructure, in accordance with the AUTOSAR compliance offered by the protocols. The gateway is delivering the data between SOME/IP and DDS nodes, assuring at the same time the interaction with the eCAL supervisor node that is placed in a separate location. Each entity is running on a separate device and the transmitted data are structured under the form of a heartbeat cyclic event. The hardware architecture can be observed in Figure 3.

With the protocol widely used in the automotive domain, for the running SOME/IP entities, the choice was processors validated by the industry and also accessible at a lower price under the form of general use platforms for development. Considering this reason, the SOME/IP entities use two types of devices:

- The first device used was the R-Car M3e Automotive System-on-Chip (SoC) from Renesas, having dual ARM Cortex-A57 processors, quad ARM Cortex-A53 processors, and the ARM Cortex-R7 Dual Lockstep processor. R-CAR M3e is described as ideal for medium automotive computing systems and it is utilized in areas such as infotainment, gateway servers, integrated cockpit applications, and others. A Linux operating system with kernel version 5.10 was used on the R-Car M3e SoC, compatible with the open-source SOME/IP implementation used in the development phase.
- The second device used for the SOME/IP entities was the Raspberry Pi 4 (R-Pi 4) based on a Broadcom BCM2711 SoC with a 64-bit quad-core ARM Cortex-A72 processor, widely used in different types of applications, including IoT and communication scenarios. A Linux operating system with kernel version 5.10.63-V7l+ was used on the R-Pi 4, the final setup offering a low cost/high performance balance.

The DDS middleware, being confirmed as a capable industrial solution in automotive and IoT/IIoT applications, offering flexibility and high performance even with low resources, was suitable for use in a device from an older R-Pi generation. R-Pi 3 uses a Broadcom BCM2837 SoC with a 64-bit quad-core ARM Cortex-A53 processor and a Raspbian operating system with kernel version 5.10.17-V7+, being suitable for the DDS applications in the current context.

For the gateway application, which represents the central part of the architecture, the selected device was R-Pi 4. The main motivation for the selected device was the compatibility of SOME/IP, DDS, and eCAL in cooperation, each one requiring a particular set of dependencies. Ubuntu 21.10 with kernel version 5.13.0-1011-raspi was used on the R-Pi 4 gateway device, the setup serving as the link between the simulated automotive infrastructure that incorporates SOME/IP and DDS, and the eCAL node more oriented to a “cloud” entity that receives automotive-specific data. The eCAL node runs on a virtualized Linux operating system, placed in a separate location, mapping to the simulated V2X scenario and acquiring the same heartbeat event that is distributed by the gateway to all interested consumers.

The presence of different high-level communication technologies in the same SOA can enhance the capabilities and offer accessible ways of adapting the architecture easily, in a modular manner. Although in general, the approach in the industry is to adopt a main protocol for all related procedures inside an architecture, the benefits of having different sets of mechanisms for a wide range of use-cases must not be ignored, especially in the IoT context. The current architectural approach of having SOME/IP as the standard automotive Ethernet-based communication solution interacting with the DDS middleware, which is compliant with AUTOSAR and also is compatible with other DDS implementations from the industrial context, expands the interoperability possibilities proportionally with the adoption of high-performance hardware and the adaptive platform. Adding to the mix a more lightweight but capable protocol, developed with automotive considerations, such as eCAL, the implementation of authentic V2X use-cases becomes feasible. One of the main goals established in the architecture definition process targeted the realistic perspective of such an SOA. The presence of different types of hardware devices and native and simulated operating systems complies with industrial trends in IoT and the automotive domain, showcasing the development struggles and achievements for upcoming communication systems.

**Figure 4:**
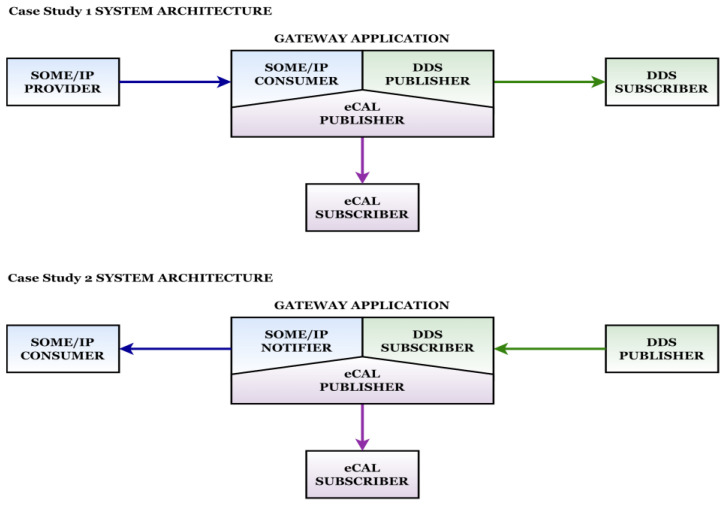
System architecture showcasing both versions of the gateway.

The conceived gateway solution delivers data between three other entities at configurable time recurrences. Besides the development of SOME/IP, DDS, and eCAL publishers and subscribers, the gateway must engage all the participants and manage the efficient distribution of the payload, considering the possible desynchronizations that can appear due to network instability or the slow reaction of the operating system for hard real-time requirements. Based on which entity is the provider of the heartbeat event, two versions of the gateway application have been defined. Each version contains the necessary subcomponents specific to each middleware in accordance with the assigned roles in the design, and each subcomponent receives and transmits the data on a separate thread. Having multiple threads associated with individual protocols allows a better observation of the recurrent transmission and possible delays or connectivity malfunctions. The data are received and delivered in an efficient way, the application responsiveness being maximized. The system architecture for both gateway versions is presented in Figure 4.

**Figure 5:**
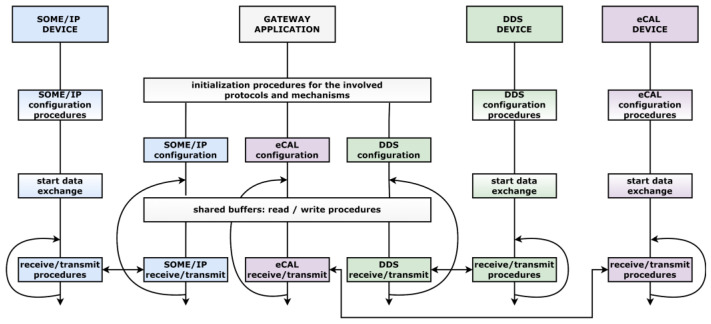
Procedure sequence for all nodes of the architecture.

The concept and development of the targeted case studies allow a clear observation regarding the procedure sequence when dealing with an SOA and with nodes with different technological origins. The procedures and the interaction between them are synthesized within our considered approach and can be observed in Figure 5.

**Figure 6:**
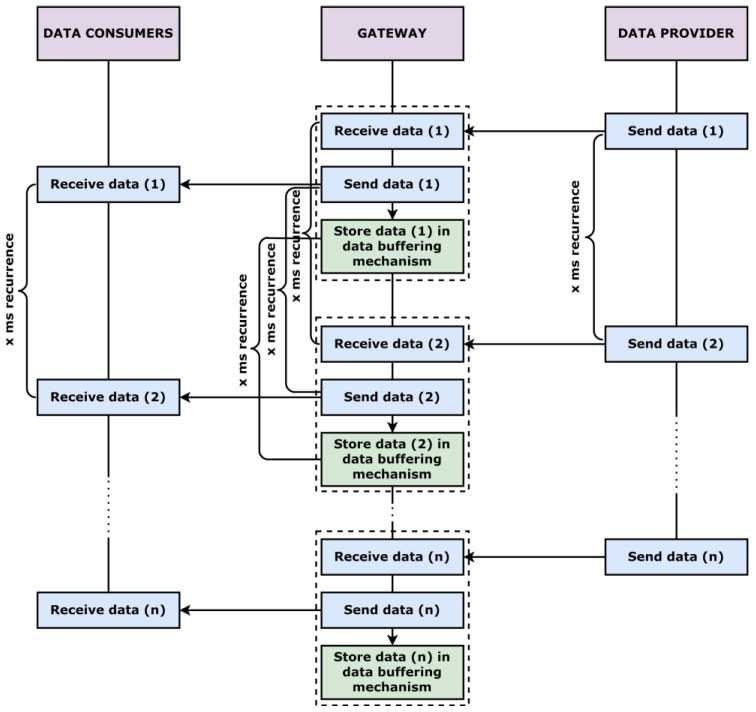
Data buffering sequence.

Objectives of case study 1 and 2 targeting the reliability and efficiency of the concept are analyzed by conceiving and developing a data buffering mechanism and a signal-generating mechanism for the distributed heartbeat event. Each mechanism provides clear results concerning the behavior of the communication infrastructure when taking into consideration real-time requirements and the impact that the network can have on the delivery of messages at fast recurrences. The data buffering capability is considered an important feature in IoT industrial communication systems. For the current scenarios, the data buffering is important for a better understanding of the obstacles that may emerge in complex SOAs. The conceived and developed data buffering process is detailed from an architectural perspective in Figure 6.

The defined architecture is feasible for data delivery in a time deterministic manner, combining devices, strategies, and technologies according to industrial scenarios. The successful interaction of the three selected protocols expands the applicability to a more complex and capable system, mapping on the IoT principles but maintaining the technological essence of the automotive field.

## 5. Case Study and Results

The current section provides insights regarding the real-time communication, evaluating the compatibility of the protocols in the defined architecture by applying methods and criteria specific to industrial scenarios. Based on the two versions of the gateway application, there are two different case studies, each presenting detailed results and observations about the system capabilities. Both case studies are simulating a V2X use-case, where SOME/IP and DDS constitute the intra-car communication infrastructure. With the gateway translating the messages for all the receivers, a connection is made with an external eCAL node. The configuration of the gateway application from one case study to the other is performed with minimal effort and no hardware architectural changes are needed, although functional changes and different roles are assigned to each node.

### 5.1. Case Study 1 and Case Study 2 Development

The main goals of the current implementations target high efficiency while the data are delivered cyclically to the consumers, assuring reliability for all the protocols even for fast recurrences. For case study 1, the gateway application is configured to facilitate the communication between a SOME/IP provider and DDS and eCAL consumers, each node being specific to a separate device. As described in the architecture section, for the SOME/IP provider, two different devices were used, validating the solution on automotive- and IoT-specific hardware and obtaining similar behaviors. From a software perspective, the portability of the SOME/IP entity confirms the potential of the technology, even if was used with an open source implementation of the protocol and not in the AUTOSAR context. The SOME/IP communication between the provider and the consumer is based on the notify–subscribe paradigm, according to the needs of the current scenario where the message is structured under the form of a heartbeat event. The receiver of the message is the gateway application, the SOME/IP subscriber subcomponent, allowing afterwards the translation of the message for the other interested nodes. The network configurations alongside other protocol-specific configurations are realized through the use of json files that are passed at runtime to the applications by the use of protocol-specific environment variables. This approach allows a separation between application logic and configuration particularities that are necessary to some extent for any Ethernet-based communication protocol, maintaining an alignment to the standard specific industrial approach, where json and arxml files are commonly used for configuration purposes. The development of the SOME/IP entities in the current design assures a reliable data provider for the current use-case, and the interaction with the other protocols does not decrease the performance.

The gateway application represents the central node of the system, communicating over three different protocols with the other nodes. The gateway is divided into three different subcomponents, each one facilitating the data delivery to the complementary node. In the current design, the subcomponents of the gateway application are represented by a SOME/IP subscriber, a DDS publisher, and an eCAL publisher. The data are received by the SOMEI/IP subscriber and afterwards are passed to the DDS publisher and prepared for delivery to the DDS consumer node. The same procedure is also executed for the eCAL subcomponent, for all the received messages. For increased efficiency, each subcomponent is running on a different thread, allowing for the gateway to receive and transmit the messages at the desired recurrence with the possibility to identify between which entities a delay might appear. From the configuration standpoint, the DDS subcomponent is initialized as a participant in a domain and assigned the publisher role. The DDS consumer node is likewise initialized as a participant in the same domain but assigned a subscriber role. The delivered message is structured as a topic, and matching operations between protocol-specific reader and writer entities are performed for identifying the producer of the data and the consumer. The DDS publisher subcomponent and the DDS consumer node are sharing the same domain, being a complete separate communication infrastructure, not available for any other node, making the communication safe and reliable, without the need to keep track of different nodes and their network particularities. The eCAL subcomponent of the gateway application represents the provider of the heartbeat signal outside of the intra-car-simulated communication infrastructure depicted by the presence of SOME/IP and DDS middleware. The eCAL protocol can be perceived as the suitable solution for V2X communication due to the minimal configuration effort needed alongside high flexibility, being developed as a straightforward way of sending messages over the network. As in the case of SOME/IP and DDS, the eCAL protocol implements the publish–subscribe paradigm, allowing for the eCAL subcomponent of the gateway application to have the role of the publisher, sending the data to the eCAL consumer node. The DDS and eCAL consumer nodes can be depicted as the final destination endpoints for the data, both interacting with the gateway application independently, receiving the heartbeat signal at the configured recurrence. With the eCAL node being considered a cloud-oriented entity, it is expected that the device to which the simulated intra-car communication structure sends the message through the gateway would be placed in a different location than the rest of the nodes. For aligning as much as possible to the principles of V2X communication, for the current study, the eCAL consumer device was completely separated from the other devices, the only link towards it being the specific subcomponent of the gateway. However, the distance did not delay the receiving process, even for very fast transmission cycles, and the eCAL consumer node managed to receive the signal without connectivity issues.

Besides the implementation of each protocol-specific entity and the gateway application, the behavioral evaluation of the designed architecture needs to comply with industrial expectations for automotive and IoT systems, considering the central gateway node as the bridge between different communication technologies applied in multiple domains. Based on such expectations, two criteria were considered, and for each one, additional mechanisms were developed, assuring a wider view upon communication capabilities in future complex use-cases. The first criterion considered was the efficiency of the receiving and transmission processes, through data buffering operations for the gateway node. With no time delivery assurances available for Ethernet communication and with possible network desynchronizations between devices, the data buffering mechanism offers a quantifiable result regarding the processed information for an individual node of the system. In comparison with the expected ideal behavior, the obtained results can determine the compatibility between different communication technologies inside the same architecture. This contributes to possible improvements for the developed software components of the system, or to possible reevaluation of the utilized operating systems and devices. The second criterion targeted the reliability of the system for receiving and transmitting events at different time recurrences, the main objective being the estimation of the feasibility level considering real-time requirements. For observing exactly how the network delays and device desynchronizations impact the system at the level of the central gateway node, the heartbeat event needed to be observed in different time deterministic scenarios. An additional mechanism was implemented, as a software subcomponent of the gateway application, which generates a precise digital signal based on the received and distributed heartbeat event. With the possibility of observing how the heartbeat event is altered at fast transmissions, the predictability of the system increases, offering a clear view of how the distributed data can be affected for Ethernet transmissions in complex architectures.

The two selected criteria can be applied at a wider scale if necessary, being relevant in the context of SOAs for both automotive and IoT scenarios. The identification of system limitations for the communication procedures is one of the most impactful objectives that must be achieved for the IoT transition. Especially in the case of field-level devices more oriented toward real-time requirements, the challenges are diverse, not related only to communication protocols but also to architectural particularities, hardware, and operating system capabilities, facilitating standards and protocols specific to the base layers of the OSI model. For the current implementation, the obtained results based on the mentioned criteria and methods offer relevant conclusions regarding the compatibility and feasibility of the utilized protocols, also validating the defined architecture.

The differences between the two case studies are represented by architectural design and role distribution among the nodes of the system. In the second case study, the provider of the information is the DDS publisher node that transmits the data cyclically to the gateway application. The subcomponents of the gateway application are also modified according to the current design, each one receiving or transmitting the heartbeat event to interested nodes. The data provided by the DDS publisher are received by a DDS subscriber subcomponent and afterwards are distributed to the SOME/IP notifier and to the eCAL publisher subcomponents. The SOME/IP notifier transmits the data to the SOME/IP consumer node, and the eCAL publisher subcomponent transmits the event to the eCAL consumer node. Both consumer nodes are final destinations of the generated heartbeat event. The simulated intra-car communication structure remains the same, composed of SOME/IP and DDS entities alongside the gateway application. Only the roles of the nodes are different, the hardware devices interacting in the same way. The V2X principles are also applied for case study 2; the eCAL consumer node simulates the interaction of intra-car communication structures with the exterior, and as in case study 1, it is placed in a different location, exchanging messages only with the complementary subcomponent of the gateway application. The gateway application maintains the approach of assigning every protocol-specific communication procedure to a separate thread, providing a detached and optimized software design that is suitable for use-cases where multiple technologies interact independently. 

An additional objective for case study 2 is to validate the case study 1 results in a slightly different context, proving that the achieved objectives and the conclusion regarding the compatibility and feasibility of the involved protocols remain the same even with modifications of the system nodes. One of the purposes of the defined architecture is to allow different configurations without the need to change the whole system. This contributes to industrial principles regarding the modular integration and modification of software components in a reliable manner, considering the same expectations in terms of efficiency and scalability. The data buffering mechanism alongside the additional subcomponent responsible for generating a digital signal based on the heartbeat event are also applied for case study 2, the expectancy being a similar behavior as for case study 1.

The gateway application remains the central node of the system, implementing multiple subcomponents that exchange information with the other nodes over three different communication technologies in an efficient and rapid manner. The applicability of the concept in different architectural alternatives confirms that the strategy of combining state-of-the-art communication technologies with different particularities can provide scalable solutions, able to satisfy various industrial demands. The case study 2 results showcase how relevant the applied criteria and methods can be. For the current system, they allow a clear perception of the possible limitations that may occur in complex scenarios which focus on multiple industrial domains.

### 5.2. Results

**Figure 7:**
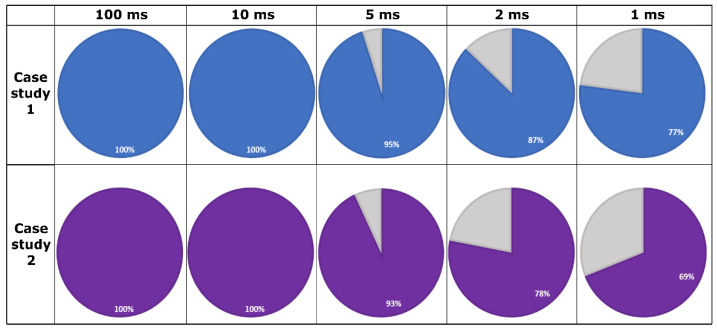
Data buffering success rate results.

**Figure 8:**
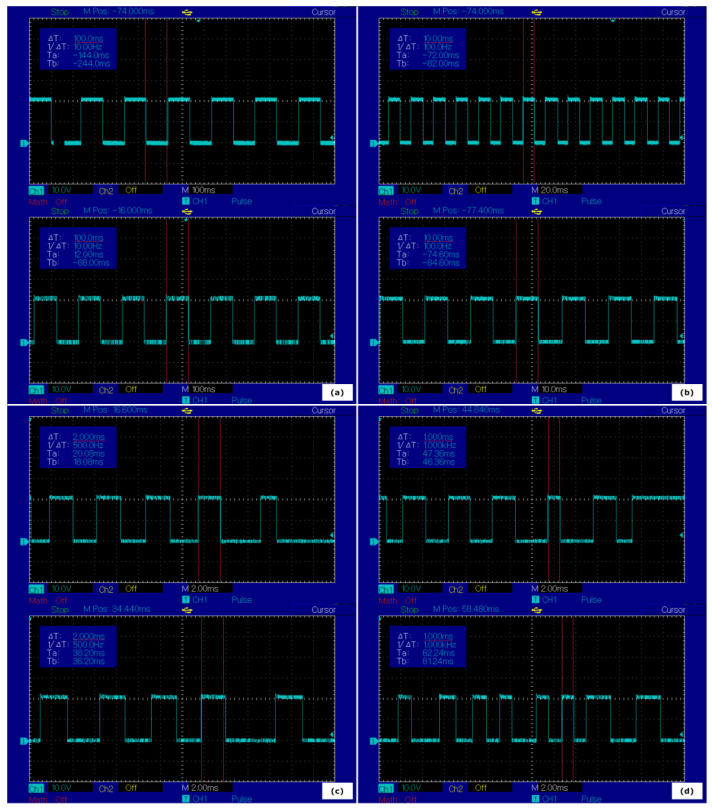
Generated digital signal based on the heartbeat event received and transmitted by the gateway application for case study 1 (above) and case study 2 (below) at (**a**) 100 ms; (**b**) 10 ms; (**c**) 2 ms; (**d**) 1 ms.

The implementation of the gateway application with all necessary subcomponents alongside all the additional protocol-specific entities was successful. The activity of each node was tested individually considering the capabilities of each protocol, assuring high interoperability even in more complex scenarios. The multithreading design of the gateway application proved efficient and feasible in the context of three separate technologies that implement a publish–subscribe pattern, the data being delivered cyclically in an automated manner. Regarding reliability and efficiency, the success rate results of the data buffering mechanism from Figure 7 applied for case study 1 and 2 can be observed in Figure 8.

The obtained results confirm the compatibility of the concept with different designs for the gateway application and with different roles assigned to the nodes of the architecture. The data buffering subcomponent alongside the signal generator subcomponent of the gateway application allowed a clear observation regarding the efficiency and reliability of the system event in the case of a different data provider and consumers. The results concern the behavior of the communication infrastructure considering real-time requirements and the impact that the network can have on the delivery of messages at fast recurrences. As expected, the efficiency of receiving and transmitting procedures decreased at high recurrences, some of the messages being lost due to network delays. The time intervals selected for observation aimed to highlight the regress from an almost certain transmission of the event, to 1 ms transmission cycles, considered a reasonable performance for the current system. Another contributing factor to less accurate transmissions is the inability of the general usage operating systems to handle operations under 10 ms and to assure the necessary responsiveness for hard real-time requirements. 

Compared to case study 1 results, the efficiency of the data buffering process decreased insignificantly for case study 2 for under 10 ms recurrences, maintaining a similar behavior for both versions of the gateway application. It is important to identify the technological and architectural limits and properly evaluate the achievable use-cases and requirements. The network capabilities, the usage of native operating systems for the devices with publishing role or mixed roles, the implementation particularities, and the multithreading design can impact the results to some extent, it being improbable to obtain a standard assessment regarding SOAs which combine multiple technologies and hardware equipment.

The second method implemented for visualizing how the distributed heartbeat event can be altered under various conditions is represented by the signal-generating mechanism as part of the gateway application. The generated results are in concordance with the data buffering success rate results, showing the reliability of the system when real-time expectations are in place. This type of practical surveillance for complex communication over Ethernet use-cases can identify clear limits for a particular system, offering the possibility to improve unfavorable aspects. The behavior of the system at various message delivery recurrences was possible by monitoring the generated digital signal based on the heartbeat event with an oscilloscope. The results can be observed in Figure 8.

The results prove that the system is capable of delivering the data in the desired time frame for all the configured recurrences. However, as expected, the sustainability decreases in a proportional manner with the increasing rate of the transmissions. The behavior matches the realistic expectations of a complex architecture with different devices and operating systems, considering also the interaction of three communication technologies, each one with significant particularities. For the less than 10 ms intervals, the irregular generated signal based on the distributed heartbeat event is easily observable, with rare accurate pulses, far from an ideal performance.

When discussing the efficiency of an Ethernet-based communication system, without dedicated standards and protocols in place, for assuring that the devices are synchronized over a common time base and each one can deliver the data accordingly to the hard real-time requirements, there are no guarantees that the transmission will be constantly performed in the desired time frame. The current results prove the efficiency of Ethernet-based communication between multiple field devices, highlight the limits of a complex system, and validate the compatibility of the protocols and the architecture by applying industrial principles from automotive and IoT fields. The interaction between various already-established technologies and architectures from different domains will be unavoidable, meaning that many challenges will derive from compatibility and efficiency issues. Identifying suitable communication protocols and architectural designs alongside limitations and improvement directions for expanding the applicability of present industrial concepts will produce reliable and scalable applications for future industrial interoperability use-cases.

**Figure 9:**
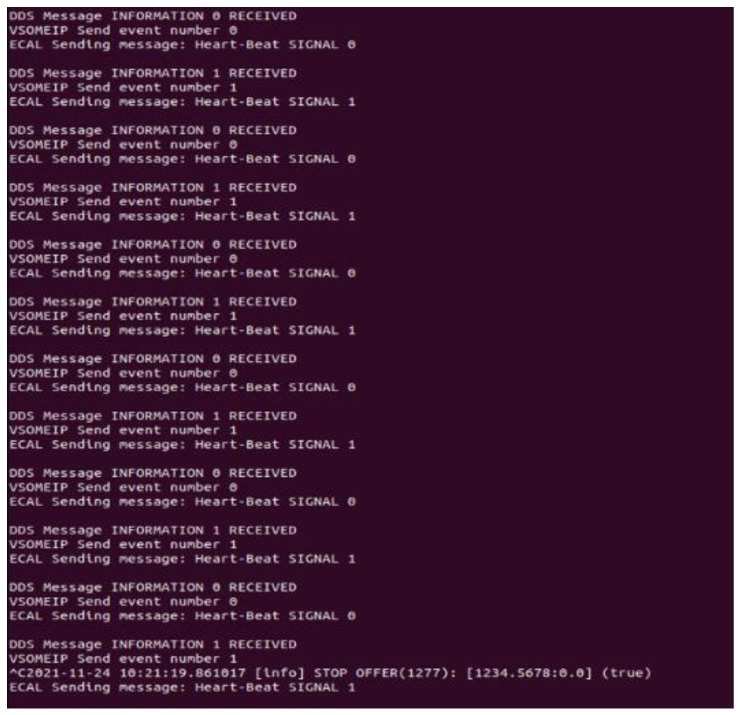
The interpreted pulse of the heartbeat event, according to receiving and transmitting sequences of the gateway application in case study 2.

Despite efficiency dropping significantly in the mentioned conditions, the interaction between SOME/IP, DDS, and eCAL cannot be considered a contributing aspect to that fact for the current implementation. The generated signal routine acknowledges the state of the received and transmitted heartbeat signals for all three subcomponents of the gateway application and generates the according pulse with high precision. For better process monitoring, another routine was developed for interpreting the heartbeat pulse for all threads of the gateway application for all receiving and transmitting sequences, making it easier to follow if the threads are synchronized and interact properly. In Figure 9, the interpreted pulse of the heartbeat event can be noticed, according to the receiving and transmitting sequences of the application for successive iterations. It reveals a correct handling of the delivered data between the separated protocol-specific threads.

The multithreading strategy for the management of the communication procedures increases the efficiency of the system and allows a clear separation between technological particularities inside the same software application. The system architecture has been validated based on similar and satisfying results in both case studies, allowing a flexible and scalable approach for dealing with multiple devices and nodes with different roles and technological origins.

With all the current work objectives accomplished, a closer examination is necessary regarding what are the most important conclusions alongside an analysis for the involved technologies and for the importance of the concept for the industry. The multi-protocol gateway managed to interface SOME/IP, DDS, and eCAL entities successfully, considering efficiency and reliability criteria and mapping the concept to a V2X communication scenario. The defined architecture was suitable for the interaction between three different communication technologies in two case studies, each one based on a different version of the gateway and with significant particularities. The results regarding the real-time behavior allowed a better understanding of the challenges concerning Ethernet communication in the context of IoT, and the methods and strategies used for generating the results can be applied at a larger scale, offering clear improvement paths. The compatibility of SOME/IP, DDS, and eCAL has been confirmed, offering an overview of the perspectives in different industrial domains and identifying certain advantages and disadvantages related to each protocol, as shown in Table 1.

Based on the current study and on the industrial context, it is clear that SOME/IP represents a reliable and efficient solution for Ethernet communication, being supported by the AUTOSAR standard and widely present in automotive applications. Being complex and suitable for multiple scenarios, the configuration process requires time and effort especially in complex SOAs, as was also concluded in the current work when compared to an IoT protocol. DDS is becoming more present in IoT industrial systems and its mechanisms offer increased flexibility and scalability. The AUTOSAR compliance also makes it suitable for automotive applications, and although for now it is not established, any possible interactions with the SOME/IP middleware in an automotive-related context represents a high interest topic of research. The eCAL middleware is not very known, but nevertheless, it offers an easy-to-use and intuitive alternative for Ethernet message delivery. Its potential is not fully determined, so the interaction with other middleware solutions will contribute to a better understanding of how it can be applied. 

**Figure 10:**
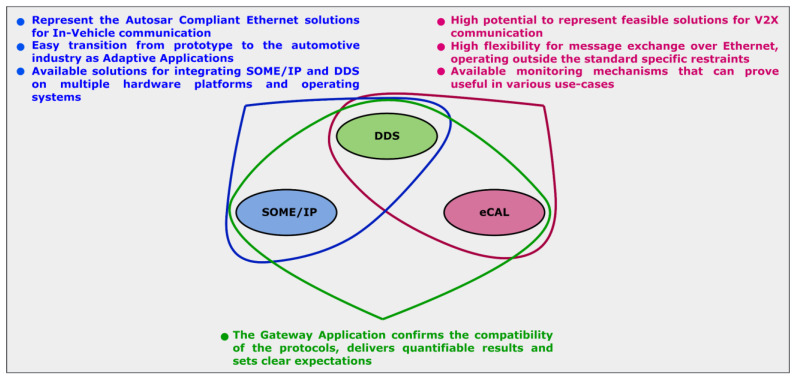
An overview of the utilized technologies.

After the analysis of the targeted communication protocols, it is important to examine the current concept in a possible industrial context. Besides the already-mentioned compliance with the automotive standard as a favoring factor, the transition of SOME/IP and DDS applications to the industry should be feasible under the form of adaptive applications, compatible nowadays with microprocessors that are established as master and zone controllers for various functional areas inside the vehicle. Being part of the adaptive platform, such applications should follow the principle of being hardware agnostic and compatible with POSIX operating systems. The current defined architecture assures such diversity for the utilized devices and operating systems. For the challenge of dealing with a high number of dependencies required by the targeted protocols of the current concept, solutions are available for industrial scenarios. The use of tailored POSIX operating systems is a common practice that became more popular in recent years, the OS being adapted to function on specific hardware devices. Such an OS can be designed to also satisfy particular necessities for the majority of the Ethernet-based protocols, assuring the compatibility between them and allowing a transition from a prototype that uses open-source libraries to an industrial product. A notable solution for tailoring POSIX OS is the Yocto project [46]. Although the SOME/IP and DDS could be established as adaptive applications in an authentic industrial scenario, the presence of eCAL and DDS allows a wider perception for the current concept when examining the industrial potential. Still considered more an IoT protocol, DDS is opening real possibilities to target V2X scenarios when functional prototypes showcase the main implications and set realistic expectations. The quantifiable results concerning the feasibility, reliability, efficiency, and real-time behavior in this case become more valuable, allowing a better comprehension regarding present and future directions. A summarized depiction concerning how the analyzed technologies and the current concept could map to the industry is shown in Figure 10.

## 6. Conclusions

The Ethernet-based communication structures represent the foundation of the concept, with efficiency and reliability under any circumstances being major objectives for any long-term solutions. The validated communication protocols offer multiple designated mechanisms for achieving the needs of the industry and are well established in particular industrial domains. The interaction between the targeted protocols is highly probable in the future. The evolution of the current systems will not be sustainable by redefining the architectures to comply with a single communication technology, but rather by an enhancement process that could facilitate the interoperability between the major industrial systems.

The current research aimed to combine three communication protocols feasible for automotive and IoT scenarios. The implemented multi-protocol gateway successfully facilitated the data exchange between different entities, expanding the applicability of the targeted technologies to a V2X scenario, confirming at the same time the compatibility between the protocols. The defined architecture assured efficient communication between multiple hardware devices, operating systems, and decoupled nodes of the system. The custom developed mechanisms based on relevant criteria offered quantifiable results concerning the real-time responsiveness of the system, defining a set of clear expectations for current intelligent communication systems. The challenges faced during the development phase were related to the integration of the selected technologies in the same architecture and in the gateway application, multiple dependencies being hard to resolve. Besides the effort to conceive and develop the solution with all the complementary entities, additional test code was designed and implemented. Individual test procedures were performed for each protocol before obtaining detailed results. The defined architecture had to anticipate system needs and minimize configuration and testing effort, following authentic industrial principles. The multi-protocol gateway is applied in a V2X concept, targeting automotive and IoT aspects of high interest for the industrial and academic communities. 

Although all the proposed objectives were achieved, and the concept is aligned with complex industrial principles and expectations, there are improvement directions that have the potential to increase the performance of the system. The adoption of time-sensitive network technology could assure a synchronized behavior of all the involved devices, guaranteeing data delivery according to the configured recurrences. The use of operating systems with specialized features for hard real-time requirements should enhance the ability to manage time-critical operations, even in the context of a complex SOA. The methods and strategies applied in both case studies allow a step-by-step addition of any improvement solutions, the results remaining quantifiable and relevant. 

The multi-protocol gateway application, as part of an SOA composed of multiple technologies, devices, and operating systems, displays communication capabilities over Ethernet considering industrial objectives and challenges. The concept is relevant for automotive and IoT domains by taking into account trends and strategies of high significance from industrial and academic perspectives.

## Figures and Tables

**Table 1:** Advantages and disadvantages related to SOME/IP, DDS, and eCAL.

Protocol	Advantages	Disadvantages
SOME/IP	AUTOSAR-compliant Validated in multiple automotive use-cases Supported on both classic and adaptive platforms Reliable and efficient	Complex configuration process
DDS	AUTOSAR-compliant Offers multiple mechanisms that assure flexibility and scalability Supported on adaptive platform Validated in multiple IoT applications and use-cases	Not established in the automotive domain, despite being AUTOSAR-compliant
eCAL	Efficient, intuitive, and easy to use for Ethernet communication scenarios Easy configuration process High potential for industrial and automotive-related use-cases	Not AUTOSAR-compliant for now Not very known Not applied to full potential in explicit technical areas

## Data Availability

Data is contained within the article.
